# Machine Learning Applied to the Search for Nonlinear Features in Breeding Populations

**DOI:** 10.3389/frai.2022.876578

**Published:** 2022-05-20

**Authors:** Iulian Gabur, Danut Petru Simioniuc, Rod J. Snowdon, Dan Cristea

**Affiliations:** ^1^Department of Plant Breeding, Justus-Liebig-University, Giessen, Germany; ^2^Department of Plant Sciences, Iasi University of Life Sciences, Iasi, Romania; ^3^Institute of Computer Science, Romanian Academy, Iasi Branch, Iasi, Romania

**Keywords:** machine learning, feature selection, linear models, genomic selection, wheat, oilseed rape

## Abstract

Large plant breeding populations are traditionally a source of novel allelic diversity and are at the core of selection efforts for elite material. Finding rare diversity requires a deep understanding of biological interactions between the genetic makeup of one genotype and its environmental conditions. Most modern breeding programs still rely on linear regression models to solve this problem, generalizing the complex genotype by phenotype interactions through manually constructed linear features. However, the identification of positive alleles vs. background can be addressed using deep learning approaches that have the capacity to learn complex nonlinear functions for the inputs. Machine learning (ML) is an artificial intelligence (AI) approach involving a range of algorithms to learn from input data sets and predict outcomes in other related samples. This paper describes a variety of techniques that include supervised and unsupervised ML algorithms to improve our understanding of nonlinear interactions from plant breeding data sets. Feature selection (FS) methods are combined with linear and nonlinear predictors and compared to traditional prediction methods used in plant breeding. Recent advances in ML allowed the construction of complex models that have the capacity to better differentiate between positive alleles and the genetic background. Using real plant breeding program data, we show that ML methods have the ability to outperform current approaches, increase prediction accuracies, decrease the computing time drastically, and improve the detection of important alleles involved in qualitative or quantitative traits.

## Introduction

Feature selection (FS) represents the identification of a subset of predictor variables that have the ability to find genetic patterns associated with a variable, in our case with a phenotypic trait. Reducing the amount of data from high-dimensional data sets, such as those we have in practical plant breeding populations, decreases computational time and can prevent overfitting and the curse of dimensionality (Guyon and Elissee, 2003). In genomics, FS allows the reduction of redundant genetic areas from the new, high-yielding genetic tools, such as single-nucleotide polyphormism (SNP) chips or genome-wide sequencing data. Moreover, many modern genomic techniques produce data that have large levels of redundancy. In this context, FS can contribute to the design of low-density SNP chips that capture most of the genetic variation necessary for practical plant breeding. In modern plant breeding programs, extracting the key features that are involved in biological processes of traits of interest and designing cost-efficient prediction tools are preferred.

Modern statistical models used for predicting phenotypes, estimating breeding values, or that deliver high rates of genetic gain over time are essential for breeding. Methods such as genome-wide association studies (GWAS) and genomic selection (GS), which use the genetic variation from markers to associate/predict phenotypic information, are frequently used in breeding programs (Bernardo, 1994; Meuwissen et al., 2001). GS is a standard tool used in breeding program nowadays as it can decrease costs by reducing the length of breeding cycles or increasing selection gain over time (Pérez-Rodríguez et al., 2012).

Machine learning (ML) algorithms could be used for genomic predictions in data sets that contain a larger number of parameters than observations, as is the case for most plant or animal breeding populations. ML implements a wide variety of techniques to identify patterns or predict characteristics in extremely large data sets. In addition to trivial examples, like unsupervised machine recognition software used to identify cats in YouTube videos, or the software “AlphaGO”, which defeated professional players in the highly complex computer game “Go” (Silver et al., 2016), ML techniques have also found their way into daily life in the form of numerous widely used personal computer software applications (e.g., Google). ML was efficiently applied, in very different research fields, to improve forecasts of major earthquakes (DeVries et al., 2018), the prediction of human population responses to toxic compounds (Eduati et al., 2015), and the prediction of drug–target interaction (Chen et al., 2018). As with suggestions made by Google and other “consumer choice” software, crop breeding shares the important objective of predicting and selecting the most favorable candidates from enormous, diffuse, and diverse data sets. Human genetics already widely exploits the two major ML branches. Firstly, supervised ML techniques help solve problems for which explanatory and response variables are available, which are commonly applied to quantitative genetics methods used for prediction, selection, and classification (e.g., the discovery of genetic factors associated with complex traits). Secondly, unsupervised procedures are used when no response variable exists. For example, population genetics often uses unsupervised procedures for problems associated with clustering of individuals and detecting genetic patterns in populations. ML-based prediction in animal or plant breeding is using high-density marker data to predict many complex traits (Gianola et al., 2011; Khaki and Wang, 2019).

In this study, we investigated several linear and nonlinear approaches for efficient FS methods combined with various parametric learners to predict important agronomical traits in a hybrid breeding population. The major objective was to identify the most efficient FS, the optimal SNP subset size, and the prediction accuracies and stabilities of the predictive models. As a benchmark, ridge regression models for GS were selected according to the methods described by Endelman (2011). Three types of FS methods were implemented: (i) linear dimensionality reduction using principal component analysis- (PCA-) based method with selection of the top PCs, (ii) nonlinear dimensionality reduction using an embedded method, such as random forest (RF), and (iii) a random selection of *n* features from the data set. The features obtained from the FS filter methods were used for the prediction of agronomical traits using ridge regression best linear unbiased prediction (rrBLUP), least absolute shrinkage and selection operator (LASSO) regression, gradient boosting machines (GBM), artificial neural networks (ANN), and Random Forest (RF).

Training and predictions were done on a spring-type *Brassica napus* population composed of 950 F1 hybrids obtained from a cross design of two mother lines (00 quality) and 475 father lines and a diversity collection of 191 wheat cultivars, registered in Europe between 1966 and 2013, tested in multi-location, multiyear field trials. Prediction accuracies were obtained as the median of the Pearson correlation between observed and predicted values in a 10-fold cross-validation (CV) scenario. The results suggested that filter methods have a positive impact on prediction accuracies for specific, in general, monogenic traits. With small- to medium-subset sizes (100–1,000 SNPs), the filter methods outperformed predictions performed with the entire data set, while decreasing drastically the computation time. The FS methods generated stable results suggesting that a low-density SNP matrix could be extracted for genome-based predictions of agronomical important traits in oilseed rape (*B. napus*) and wheat (*Triticum aestivum*).

## Materials and Methods

### Experimental Phenotype Data

Phenome data were generated for a population of 950 F1 hybrids obtained by crossing between two male sterile testers (MSL-T1 and MSL-T2, NPZ Lembke, Hohenlieth, Germany) and a diverse population of 475 spring-type “00” *B. napus* cultivars (Jan et al., 2016). F1 hybrids were tested at four different locations in Europe using non-replicated trails. Six important agronomical traits were investigated: seed yield (SY, in dt/ha), oil yield (OY, in dt/ha), seed oil content (SOC, in percentage of volume per seed dry weight), seedling emergence (SE, visual observation in a 1–9 scale), and days of flowering (DTF, number of days from sowing until 50% flowering plants per plot). We used phenotype data as predictors, which were subjected to a restricted maximum likelihood model (REML) and generated best linear unbiased estimate (BLUE).

For the diversity wheat panel, field trials included 191 cultivars tested in six locations over the growing seasons 2014–2015 and 2015–2016 (Voss-Fels et al., 2019). Moreover, the selected cultivars were analyzed in three different cropping intensities, designated as HiN/HiF (220 kgN ha^−1^ mineral fertilizer, full intensity of fungicides, insecticides, and growth regulators), HiN/NoF (220 kgN ha^−1^ mineral fertilizer, no fungicides), and low nitrogen inputs and no fungicide (LoN/NoF; 110 kgN ha^−1^ mineral fertilizer, no fungicides, insecticides, and growth regulators) treatments. For the purpose of this study, we selected the following traits for further investigations: grain yield LoN/NoF, grain yield HiN/HiF, and grain yield HiN/NoF.

### SNP Genotyping

All founder lines from the *B. napus* panel were tested using the Brassica 60k SNP Infinium consortium array (Illumina, Inc., San Diego, CA, USA). Genomic DNA was obtained using the leaf material collected after seed sowing and extracted with the BioSprint 96 extraction robot (Qiagen, Hilden, Germany). DNA quality and quantity were evaluated using a Qubit 2.0 fluorometer (Life Technologies, Darmstadt, Germany) and by gel electrophoresis. In total, 20 ng/μl of DNA per samples was sent to TraitGenetics GmbH (Gatersleben, Germany) for genotyping using the Brassica 60k SNP Infinium array (Illumina Inc., San Diego, CA, USA). The basic local alignment search tool (BLAST) was used to align SNPs to the *B. napus* cv. Darmor-*bzh* v4.1 reference genome (Chalhoub et al., 2014), according to the protocol described by Jan et al. (2016). Further investigations used 28,086 single-position SNPs for the genotyped material (Supplementary Table S1).

All wheat cultivars from the panel were genotyped with a 15K SNP Illumina Infinium iSelect genotyping platform. Raw SNP data were filtered to remove markers with more than 10% missing values and more than 5% minor allele frequency (MAF). Physical genetic SNP marker positions in the wheat genome were obtained by blasting to the genome sequence assembly for the bread wheat cultivar Chinese Spring (IWGCS Reference Sequence v1.0; https://wheat-urgi.versailles.inra.fr/Seq-Repository/Assemblies) and filtered based on quality control. For further analysis, 8,710 high-quality, polymorphic SNP probes were given (Supplementary Data File S1, Voss-Fels et al., 2019).

### F1 Hybrid Genotyping for the Spring-Type “00” *B. napus*

Single-nucleotide polyphormism marker profiles of the parental lines (pollinators and male sterile mother lines) were used to construct an *in silico* hybrid genotypes for all potential hybrid combinations, as described by Werner et al. (2018). Bi-allelic SNP markers were encoded into a 0, 1, and 2 matrices and a randomly selected allele was used as the reference at a given locus. If both parental genotypes were, at the specific locus, homozygous for the same allele, then the hybrid genotype was encoded as 0 or 2, and if they were homozygous for a different allele, then 1 was used. Heterozygous SNP from parental lines were encoded into a 0.5, 1, or 1.5 in hybrid genotypes, assuming a probability of 0.5 that an allele is inherited by an offspring from one parent. Even if it is uncertain which of the two potential alleles was transmitted in the progenies, we can assume a ratio of 50–50% of both paternal alleles to be present in a homogenous F1 hybrid (Supplementary Table S2).

### Feature Construction and Dimension Reduction

Redundant features from the input data sets can influence predictions using ML, therefore the input data should be reduced or summarized into a smaller size of features. Moreover, extraction of important features could also help understand better the input data. These approaches are known as feature learning methods that resolve the problem of setting peculiar to vectors (Mamitsuka, 2018). Therefore, feature learners aim at transforming the input data into learnable (reduce large or redundant data and make ML models run more efficiently) and interpretable (make the point of data comprehensible and interpretable) features.

Trait association and variable selection algorithms include variable ranking to make the breeding efforts and genetic-based selection simpler, scalable, and with an increase success rate. Moreover, variable importance ranking may prove to be a lucrative step in building efficient predictions.

In our approaches, we selected the following approaches for feature reduction:

(i). *Dimensionality reduction* to generate a small number of features that confidently characterize the original data set, and therefore containing the relevant properties embedded in the input data.
  In this scenario, all input data points are discarded and replaced with new features, making ML models more feasible, and results interpretable. For this paper, we selected PCA as a method for dimensionality reduction. In the literature, many other methods are available, such as Laplacian eigenmaps, multidimensional scaling (MDS), or locally linear embedding (LLE) (Mamitsuka, 2018).
Principal component analysis-based features are generated by linear combinations of features from the original data set, so that the new features explain the data visually. Let *w*_*i*_ represents the weight of the input feature *X*_*i*_:

$$\begin{array}{l} w_{1}X_{1}{+w}_{2}X_{2}+\ldots +w_{n}X_{n}=wX, \end{array}$$

where *w* is a numeric vector that has the same length as the number of features in the input data *X*. For each case, *X* can be projected into one value by *Xw*, which transforms the points on one-dimensional axis, solving the eigenvalue problem *X*^*T*^ *X* and obtaining eigenvectors, or principal components (PCs). PCs are ordered according to the eigenvector and account for a level of variance in the original data set.
*Principal component analysis data reduction* identifies the direction of the maximum variance in high-dimensional data and projects it onto a new set of coordinates (PCs).

$$\begin{array}{r} X=[X_{1},X_{2},X_{3},\;\ldots ,X_{n}],\;X\;\in R^{n}\\ wX,\;w\in R^{n\;x\;k}\\ z=[z_{1},z_{2},z_{3},\;\ldots ,z_{k}],\;z\;\in R^{k} \end{array}$$

1. Selection of *k* eigenvectors for the *k* largest eigenvalues (*k* ≤ *n*).
2. Construct the *w* matrix for the top *k* eigenvectors.
3. Transform the *n*-dimensional data set *X* using the *w* matrix to obtain the new *k*-dimensional features (PCs).

Implementation was performed using the “*prcomp()”* function implemented in R code, R version 3.6.2 (R Core Team, 2013). The same R code version was used for all other analysis.
(ii). *Feature selection* of only important features out of the original data sets and discarding the unselected ones, making our data sets for the learners smaller and easily understandable.
In this scenario, all input data points are analyzed at the same time and non-informative ones were discarded. For this paper, we selected a tree-based approach using RF as a method for FS.
Random forests are comprised of trees, as base learners, and classified as bagging ensembles. For RF, feature importance could be measured by considering, in the model, the out-of-bag (oob) instances of each tree, known as permutation importance or by considering the node impurity of a tree, or impurity filtering that uses the Gini index (Izenman, 2013).
The importance of the variables, based on permuting oob data, was calculated using the R package “randomForest” functions “randomForest,” and “importance (type=1)” (Breiman, 2001).
(iii). *Random selection* of 1,000 features from the original data set (random). As there is a high probability of a random SNP marker to be associated with a specific trait, we shuffled the SNP alleles and kept the MAF for each selected marker. This approach generates a true random marker selection matrix and removes all potential SNP alleles linked with the trait of interest. Moreover, a new marker matrix was generated that allows us to include an extra level of testing for model overfitting.

### Linear and Nonlinear Genomic Learning Models

In this paper, the following learners were selected for predicting trait-genotype breeding values: rrBLUP, LASSO, GBM, ANN, and RF.

### Ridge Regression Best Linear Unbiased Prediction

Genomic prediction accuracies were estimated using the rrBLUP model described by Endelman (2011) and Endelman and Jannink (2012) assuming the same distribution of marker effects across the whole genome. The following equation should be interpreted as a mixed linear model:


$$\begin{array}{l} y_{j}=\mu_{j}+\sum_{k=1}^{p}x_{jk}\beta_{k}\delta_{k}+e_{j} \end{array}$$

where *y* is a *p* ×1 vector of phenotype; μ is the overall mean; *x*_*jk*_ is the *k*th marker for individual *j*. We assume that δ_*k*_ = 1 for all *k*, overall mean (μ) is a fixed effect, and β, *e* are random effects.

Implementation was performed using the “caret” R package (Kuhn, 2008). The following parameters were used: function “train,” method = “glmnet,” tuneGrid = expand.grid (α = 0, λ = 0.01).

### Least Absolute Shrinkage and Selection Operator

The LASSO approach aims to archive a sparse solution *via* an additional penalty (*l*_1_), while *ridge regression* uses another penalty (*l*_2_) to deal with many predictors that have nonzero coefficients **(**Tibshirani, 1994). One could consider that both LASSO and ridge regressions are special cases of the Elastic Net, which uses both *l*_1_ and *l*_2_ penalty and a parameter between them (Zou and Hastie, 2005).

Implementation was performed using the “caret” R package (Kuhn, 2008). The following parameters were used: function “train”, method= “glmnet,” tuneGrid=expand.grid (α = 1, λ = 0.01).

### Gradient Boosting Machines

Gradient boosting machines are a prediction method that represents an ensemble of weak prediction models, normally used for its fast and easy computational leaning and improved predictive performance over stand-alone algorithms (Mason et al., 1999). The model construction is performed in a stage-wise fashion and generalized by an arbitrary differentiable loss function. The implementation was performed using the “h2o.gbm” function implemented in the R package H2O.ai (2021), H2O version 3.32.0.4.

Default settings were used for the GBM analysis, with the hyper-tuned parameter being “ntrees = 1,000, sample_rate = 0.7.”

### Artificial Neural Network

Artificial neural networks are an extension of the fundamental principle of the *Perceptron* model, which is trained on gradient data using backpropagation (Rosenblatt, 1958). Hastie et al. (2009) introduced a multiple-layer feed-forward neural network concept, and it can be seen as a multiple-step regression that extracts linear combinations of the input data in the hidden layers and model then in the output layer. For genomic prediction, in the hidden layer of an ANN, the covariates (e.g., genetic markers) are linearly combined with a weight vector and an intercept that represents the number of neurons. This linear combination is converted using an activation function, $\int_{t}(\;),$ so that it generates the output of the hidden neuron for genotype *X*_*j*_.

We used an ANN with two hidden levels, epochs = 60, and “Tanh” as the activation function, in the “h2o.deeplearning” function implemented in the R package H2O.ai (2021).

### Random Forest

*Random forest* regression was used to investigate the nonlinear combinations of variables and detect the complex interactions among variables. Variable importance was determined using a wrapper algorithm build in the “h2o.randomForest” function from the R package H2O.ai (2021).

Default settings were used for the RF analysis, only hyper-tuned parameter being “ntrees = 1,000.”

### Model CV

The training population (TP) for all selected models was set up at 80% of the total F1 hybrids in the given data set, genotype, and phenotype. At the beginning of each run, the data set was divided into a random 80% TP containing both genotype and phenotype data, and 20% validation population (VP), containing only genotype data. The prediction population (PP) contained only SNP data with no consideration of phenotype values. To evaluate the prediction accuracy of the models, we conducted a 10 times CV using the entire data set. For each CV, the 80% TP and the 20% VP were recalculated, and the training-validation cycle was repeated independently 10 times for each FS method. Models trained using only 80% of the input data, were used to calculate the predictive values using the 20% validation subset of the input data. The generated results were used to assess the prediction accuracy, using Pearson's correlation coefficients (*r*) and root-mean-square error (RMSE) between the predicted and observed phenotype data from the PP.

**Figure 1:**
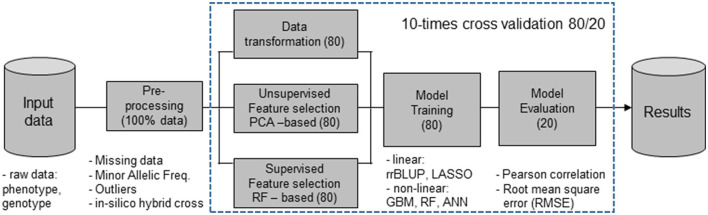
Machine learning (ML) workflow that include feature selection (FS) methods [principal component analysis (PCA)], nonlinear dimensionality reduction [random forest (RF)], and random selection using 80% of the input data set, while the evaluation of prediction accuracies was done with the remaining 20%, the validation populations (VPs). Features obtained from the FS filter methods were combined with ridge regression best linear unbiased prediction (rrBLUP), least absolute shrinkage and selection operator (LASSO) regression, gradient boosting machines (GBM), artificial neural networks (ANN), and RF predictors.

Figure 1 describes the proposed models for FS and predictors used for the evaluation of the prediction accuracies.

#### Computer Architecture

All data analyses were performed on an Intel(R) Core(TM) i9-10900 CPU @ 2.80GHz, with 64 Gb RAM, system type: 64-bit operating system, display: NVIDIA GeForce RTX 2070 SUPER, storage: 1 Tb.

## Results

### Feature Construction and Space Dimensionality Reduction

The selection of a subset of the original data set, through dimensionality reduction or FS, may have many advantages in various applications due to reduced costs related to storage and data processing. ML algorithms have the ability to design the most appropriate representation of a given data set; therefore, in many cases improved performances are observed when models use features derived from the original set (Guyon and Elissee, 2003). However, detecting the optimal feature reduction and including in the model of the key representation of the input data are specific for a given application. Therefore, in ML, there are a large number of feature construction methods, such as clustering, linear transformations [PCA/singular value decomposition (SVD)], spectral transformations, convolutions of kernels, etc.

For each FS method, 10 arbitrary selected subsets that contain 80% of the input data are selected and CV is performed in the 10-fold prediction performances of the rest 20% to evaluate the prediction accuracy for each individual feature filter method-learner combination.

The first FS method, PCA-based dimensionality reduction, used only the top 100 PCs that explained 85.8% of the genetic variance present in the SNP marker matrix (Supplementary Figure S1). The selected features were converted into input data for the selected learners and used in further analysis.

The RF-based FS investigated the number of features that have an importance score, according to the “mean decrease in accuracy,” as it has superior meaning for measuring feature relevance and can extract the optimal subset of features and reduce noise from biological data (Menze et al., 2009). In total, we selected the top 1,000 features that contributed most to the trait-marker using the RF-FS algorithms.

Modern algorithms have the possibility to perform reliable trait-genotype association, through GWAS and rank variables according to their importance for breeding objectives. These genomic-based selection algorithms, such as GS, are capable of fast and easy scalable data analysis that generates increased rates of genetic gain. Up to date, several papers described the utility of variable selection and ranking prior to the use of learners and performing predictions. A microarray analysis for the development of drug leads and identification of gene–protein interactions are some examples of successful ranking-based FS used in prediction (Guyon and Elissee, 2003). Feature importance can distinguish among redundant data entries in the input data set and key factors that influence the trait of interest and genetic marker associations.

### Learners' Prediction Accuracies

**Figure 2:**
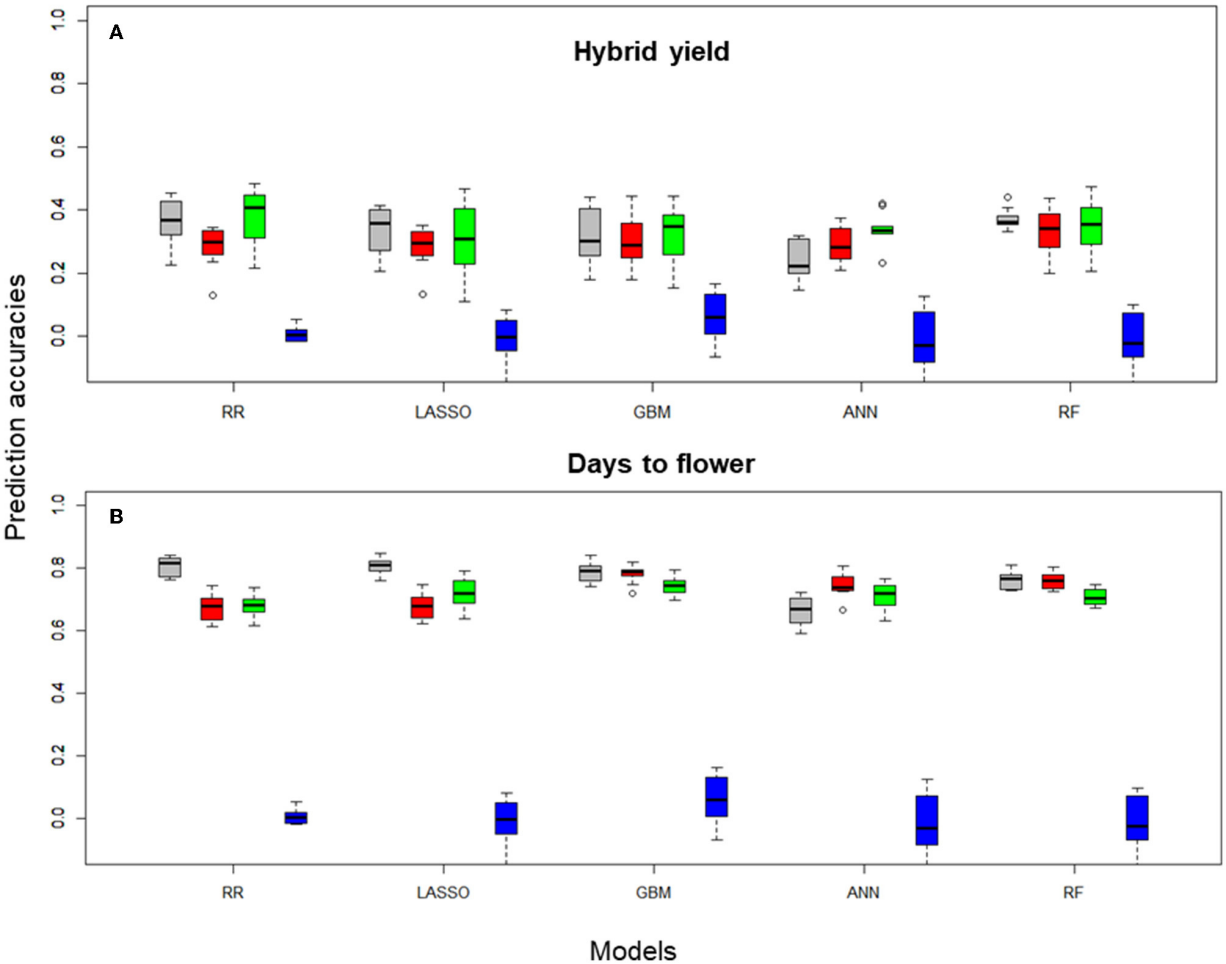
Boxplots for **(A)** hybrid yield and **(B)** days to flower predictions of the *B. napus* data set obtained on 10-fold cross-validation (CV) sets, using Pearson correlations, with rrBLUP, LASSO, GBM, ANN, and RF, with 100, and 1,000 SNP subsets selected with different filter methods and the entire SNP data set (colored in gray). Filter methods: principal component analysis 100 PCA (red), 1,000 RF (green), 1,000 RS (blue), and 14,718 (gray, no FS using the total number of markers after QC).

Prediction accuracies were generated by CV of each FS method using the corresponding input data and on an individual trait basis. Figure 2 shows the boxplots for hybrid yield performance and days to flower data obtained in the 10 CV scenarios for rrBLUP, LASSO, GBM, ANN, and RF learners with preselection of SNPs performed using the different FS methods (first 100 PCs, top 1,000 high importance SNPs for the RF-based selection, and 1,000 randomly selected SNP markers).

Hybrid yield predictions performed with rrBLUP using the entire data set generated a median Pearson correlation index of 0.30, which was not significantly different from predictions obtained with PCA-based reduction or RF-based FS. However, statistical differences were obtained between the PCA and RF reduction methods (*p* = 0.01, Figure 2A, Table 1), and among all methods and the random selection of markers. For the LASSO, GBM, and RF learners, no statistical differences were observed between the data generated with the entire set and PCA or RF-based FS. Interestingly, for the ANN learner, there is a statistical difference among predictors that use the entire SNP data set and the RF-based FS (0.01, Table 1).

**Table 1:** Median accuracy of cross-validation (CV) results using the Pearson correlation coefficient, over all pairs of feature scores in the 10-outer training sets obtained with different filter methods for feature selection (FS), of hybrid seed yield (SY), and days to flower for the *B. napus* panel and grain yield under three management practices [HiN HiF, HiN NoF, low nitrogen inputs (LoN), and no fungicides (NoF)] for the 191 wheat cultivars.

**Population**	**Trait**	**Filter**	**Learner**	**Subset size**	**Median accuracy (Pearson's correlation)**
*Brassica napus* (F1)	Hybrid yield	-	rrBLUP	1,4718	0.3674
			LASSO	1,4718	0.3566
			GBM	1,4718	0.3019
			ANN	1,4718	**0.2224**
			RF	1,4718	0.3603
		PCA	rrBLUP	100	0.2964
			LASSO	100	0.2954
			GBM	100	0.2878
			ANN	100	0.2810
			RF	100	0.3401
		RF	rrBLUP	1,000	**0.4055**
			LASSO	1,000	0.3089
			GBM	1,000	0.3478
			ANN	1,000	0.3349
			RF	1,000	0.3525
		random	rrBLUP	1,000	0.0047
			LASSO	1,000	−0.0021
			GBM	1,000	0.0596
			ANN	1,000	−0.0303
			RF	1,000	0.0244
	Days to flower	-	rrBLUP	14,718	**0.8164**
			LASSO	14,718	0.8089
			GBM	14,718	0.7932
			ANN	14,718	**0.6712**
			RF	14,718	0.7658
		PCA	rrBLUP	100	0.6795
			LASSO	100	0.6791
			GBM	100	0.7894
			ANN	100	0.7388
			RF	100	0.7600
		RF	rrBLUP	1,000	0.6810
			LASSO	1,000	0.7202
			GBM	1,000	0.7437
			ANN	1,000	0.7184
			RF	1,000	0.7056
		random	rrBLUP	1,000	0.0048
			LASSO	1,000	0.0022
			GBM	1,000	0.0597
			ANN	1,000	0.0303
			RF	1,000	0.0245
*Triticum aestivum*	Grain yield (LoN.NoF)	-	rrBLUP	8,630	0.5588
			LASSO	8,630	0.5640
			GBM	8,630	**0.5430**
			ANN	8,630	0.5496
			RF	8,630	0.6018
		PCA	rrBLUP	100	0.6849
			LASSO	100	0.6865
			GBM	100	0.5274
			ANN	100	**0.6921**
			RF	100	0.5530
		RF	rrBLUP	1,000	0.6686
			LASSO	1,000	0.5725
			GBM	1,000	0.5922
			ANN	1,000	0.5987
			RF	1,000	0.6112
		random	rrBLUP	1,000	0.0015
			LASSO	1,000	0.0803
			GBM	1,000	0.0046
			ANN	1,000	0.0230
			RF	1,000	0.0299
	Grain yield (HiN.HiF)	-	rrBLUP	8,630	0.8003
			LASSO	8,630	0.7279
			GBM	8,630	0.7280
			ANN	8,630	0.7829
			RF	8,630	0.7289
		PCA	rrBLUP	100	0.7812
			LASSO	100	0.7820
			GBM	100	0.7423
			ANN	100	0.7788
			RF	100	0.7314
		RF	rrBLUP	1,000	0.8030
			LASSO	1,000	**0.7131**
			GBM	1,000	0.7361
			ANN	1,000	**0.8030**
			RF	1,000	0.7579
		random	rrBLUP	1,000	0.0007
			LASSO	1,000	0.0050
			GBM	1,000	0.0381
			ANN	1,000	0.0537
			RF	1,000	0.0021
	Grain yield (HiN.NoF)	-	rrBLUP	8,630	**0.5709**
			LASSO	8,630	0.5047
			GBM	8,630	0.5226
			ANN	8,630	0.4482
			RF	8,630	0.4482
		PCA	rrBLUP	100	0.5472
			LASSO	100	0.5474
			GBM	100	0.4421
			ANN	100	0.5150
			RF	100	0.4317
		RF	rrBLUP	1,000	0.5362
			LASSO	1,000	**0.3712**
			GBM	1,000	0.4844
			ANN	1,000	0.4850
			RF	1,000	0.5349
		random	rrBLUP	1,000	0.0231
			LASSO	1,000	0.0050
			GBM	1,000	0.0014
			ANN	1,000	0.0803
			RF	1,000	0.0109

Ridge regression best linear unbiased prediction, LASSO, GBM, ANN, and RF combined with different filter methods showed a similar pattern and comparable prediction performance. Statistical differences were observed between rrBLUP (all SNPs) and ANN (all SNPs) with a value of *p* = 0.04 and rrBLUP (RF-FS) and ANN (all SNPs) with a value of *p* = 0.01 (Supplementary Table S3). In this comparison, random-selected markers were not included.

For a less complex trait, such as days to flowering, predictions performed with rrBLUP using the entire data set generated a median Pearson correlation index of 0.67, significantly different from predictions obtained with PCA-based reduction or RF-based FS (*p* < 0.001). However, no statistical differences were obtained between the rrBLUP_PCA and rrBLUP_RF reduction methods (Figure 2B, Table 1). A similar pattern was recorded for the LASSO combined with FS methods. Moreover, among all nonlinear learners (GBM and RF) methods and PCA, RF-based selection methods, no statistical significance was observed, suggesting that in the case of nonlinear predictors, the entire data set-based predictions performed similarly to the feature selected markers. In addition, for ANN, we observed a statistical difference among the entire data set and the PCA FS methods (*p* < 0.05) (Supplementary Table S3).

The analysis of the mean of the Pearson correlation over all pairs of feature scores in the 10 CV training sets obtained with different filter methods for FS showed the highest mean, for hybrid yield, using the RF-FS method, and rrBLUP learner, namely, 0.4055. The lowest PC mean for hybrid yield was observed for predictions using all data and the ANN learner, namely, 0.2224. For days to flowering, the highest PC mean was observed for rrBLUP using all data and the lowest for ANN using all data, namely, 0.8164 and 0.6712, respectively.

Interestingly, nonlinear learners (GBM, ANN, and RF) outperformed, on average, linear learners (rrBLUP and LASSO) when the selection of relevant features was introduced in predictions across training sets. Tree-based methods (GBM and FR) exhibited a high prediction accuracy when combined with PCA-based dimensionality reduction and RF-FS of the input data. PCA-based dimensionality reduction combined with GBM generated a 0.7895 prediction accuracy, while for ANN, 0.7388 and RF with 0.7600. For rrBLUP and LASSO combined with PCA filter methods, the prediction accuracies were at 0.679, statistically different from nonlinear learners (Table 1).

Similar analyses for the prediction accuracy were performed for all other traits (OY, SOC, and SE) and comparable results were obtained. Corresponding tables and figures are available in the Supplementary Table S4).

**Figure 3:**
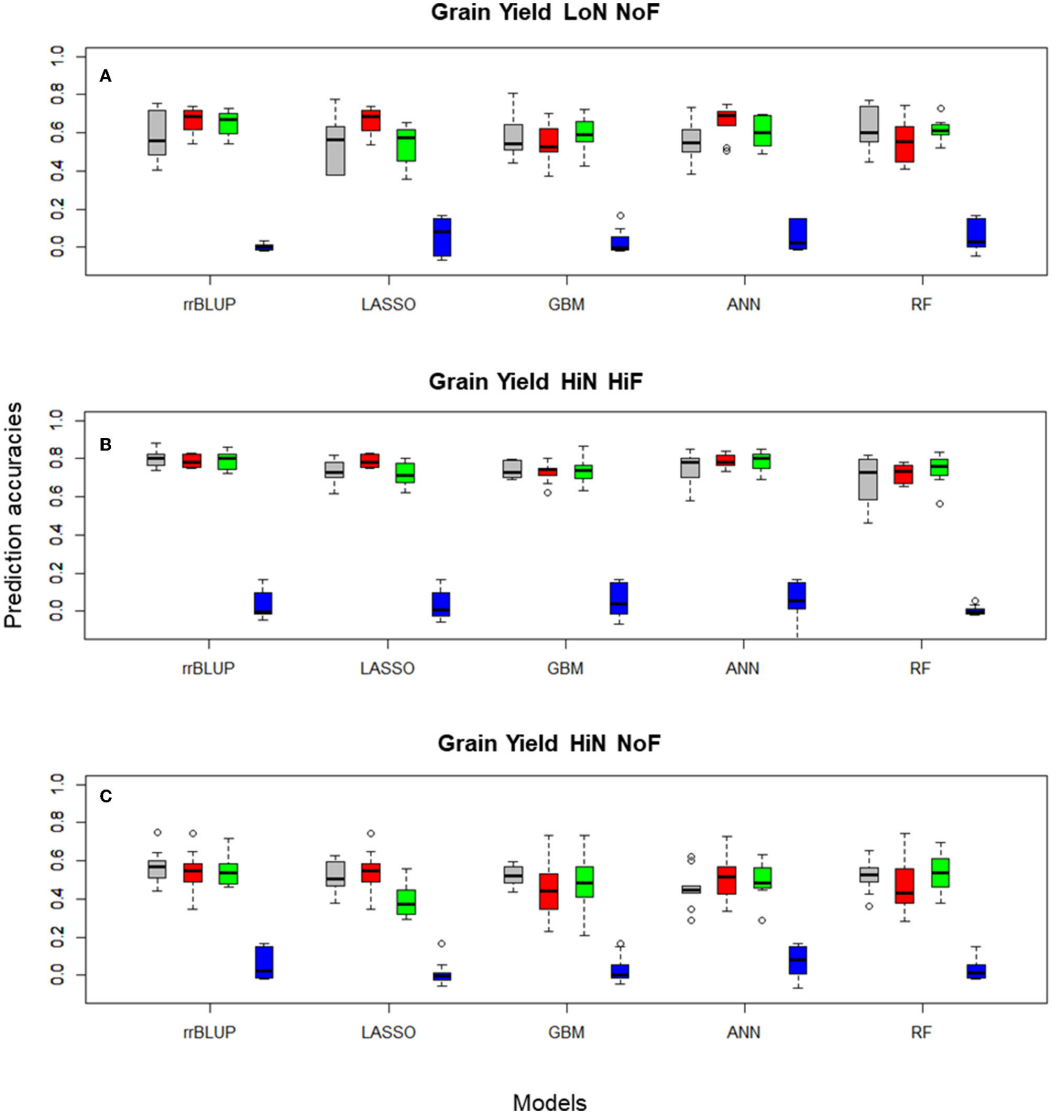
Boxplots for wheat **(A)** grain yield LoN/NoF, **(B)** grain yield HiN/HiF, and **(C)** grain yield HiN/NoF predictions of the *Triticum aestivum* 191 commercial cultivars obtained in 10-fold cross-validation (CV) sets, using Pearson's correlations, with rrBLUP, LASSO, GBM, ANN, and RF, with 100, and 1,000 SNP subsets selected with different filtering methods and the entire SNP data set (colored in gray). Filtering methods: principal component analysis 100 PCA (red), 1,000 RF (green), 1,000 RS (blue), and 14,718 (gray, no FS using the total number of markers after QC).

For the diversity panel that includes 191 commercial wheat cultivars, we observed a clear improvement in prediction accuracies when FS models were implemented (Table 1), especially for studies conducted under LoN/NoF. In this scenario, PCA-based FS showed the highest median prediction accuracy, of 0.692 for ANN, while using the entire data set the accuracy was 0.549, for the same predictor. Similar patters can be observed for all FS-learner combinations (Figure 3).

In terms of computing time, PCA-based data reduction outperformed all other FS filter methods, reducing the computing time, for all feature identification methods and learners, from 1,200 min (when using the entire SNPs data set), to 6.7 min, an approximately 200 × decrease for hybrid yield predictions using the *B. napus* population. Similar results were observed for wheat grain yield LoN/NoF predictions using PCA-based FS, which reduces the processing time from 103.2 to 3.1 min, a 33 × decrease. This trend was observed for all other traits investigated. Similarly, RF-based FS decreased the computing time by approximately 10 ×, from 1,200 to 139.7 min for hybrid yield predictions using all learners. In general, FS reduces considerably the processing time, while generating similar or improved prediction accuracies (Table 2).

**Table 2:** Processing time for filter methods, principal component analysis-(PCA-) based data reduction and random forest-(RF-) based FS, combined with linear and nonlinear learners, ridge regression best linear unbiased prediction (rrBLUP), least absolute shrinkage and selection operator (LASSO), gradient boosting machines (GBM), artificial neural networks (ANN), and RF.

**Population**	**Trait**	**Filter method**	**Learner**	**Computing time** **(min)**
*Brassica napus*	Hybrid yield	-	rrBLUP, LASSO, GBM, ANN, RF	1,200
		PCA	rrBLUP, LASSO, GBM, ANN, RF	6.7 (200 ×)
		RF	rrBLUP, LASSO, GBM, ANN, RF	139.7 (10 ×)
		random	rrBLUP, LASSO, GBM, ANN, RF	39.3 (30 ×)
	Days to flower	-	rrBLUP, LASSO, GBM, ANN, RF	749.9
		PCA	rrBLUP, LASSO, GBM, ANN, RF	7.4 (100 ×)
		RF	rrBLUP, LASSO, GBM, ANN, RF	135.7 (6 ×)
		random	rrBLUP, LASSO, GBM, ANN, RF	78.7 (10 ×)
*Triticum aestivum*	Grain yield LoN/NoF	-	rrBLUP, LASSO, GBM, ANN, RF	103.2
		PCA	rrBLUP, LASSO, GBM, ANN, RF	3.1 (33 ×)
		RF	rrBLUP, LASSO, GBM, ANN, RF	15.2 (7 ×)
		random	rrBLUP, LASSO, GBM, ANN, RF	19.6 (5 ×)
	Grain yield HiN/HiF	-	rrBLUP, LASSO, GBM, ANN, RF	97.5
		PCA	rrBLUP, LASSO, GBM, ANN, RF	3.2 (32 ×)
		RF	rrBLUP, LASSO, GBM, ANN, RF	15.3 (6 ×)
		random	rrBLUP, LASSO, GBM, ANN, RF	19.7 (5 ×)
	Grain yield HiN/NoF	-	rrBLUP, LASSO, GBM, ANN, RF	97.4
		PCA	rrBLUP, LASSO, GBM, ANN, RF	3.1 (31 ×)
		RF	rrBLUP, LASSO, GBM, ANN, RF	15.2 (6 ×)
		random	rrBLUP, LASSO, GBM, ANN, RF	21.2 (5 ×)

## Discussions

Diversity panels and new breeding populations are traditionally a source of novel allelic diversity and are at the core of selection efforts for elite material. Finding rare diversity requires a deep understanding of biological interactions among the genetic makeup of one genotype and its environmental conditions. However, most modern breeding programs still rely on linear regression models to solve this problem, generalizing the biological problem into an infinitesimal model (Fisher, 1919).

However, the recent success of ML techniques has boosted researchers to also use in modeling breeding methods that identify relevant patterns in the input data and perform deep data mining.

Human genetics already widely exploits the two major ML branches, using supervised or unsupervised leaning algorithms. ML techniques help solve problems for which explanatory and response variables are available, which are commonly applied to quantitative genetics methods used for prediction, selection, and classification (e.g., the discovery of genetic factors associated with complex traits). For example, population genetics often uses unsupervised procedures for problems associated with clustering of individuals and detecting genetic patterns in populations. Modern ML models use a wide range of statistical approaches to learn from the input data, or the training data, and predict in further genotypes. ML algorithms are extremely useful when dealing with large heterogeneous data sets (Collins and Yao, 2018), such as those commonly found in plant breeding populations.

Recently, applying FS techniques in data analysis and bioinformatics has started to gain momentum and FS becomes a prerequisite for building prediction models that have increased accuracies. The high dimensionality present in modern biological data, as genomic sequence analysis, SNP chip arrays, or hyperspectral phenomics, needs tools for a better understanding of the underling genetic mechanisms and identify patterns, sometimes in the noise, that are correlated with a specific trait (Yoosefzadeh-Najafabadi et al., 2021). An efficient feature construction method should represent the best reconstruction of the input data set, which usually triggers an increased efficiency in prediction. Identification of the most appropriate representation of the input data is an unsupervised leaning problem, due to its demand for data compression, while better prediction performance is a supervised learning problem. In general, unsupervised FS methods are less prone to overfitting (Guyon and Elissee, 2003), and have the ability to improve predictions, while discarding redundant data points.

Processing of high-dimensional data requires large computational power, while it may overfit a model and generate poor prediction. In the literature, several authors compared the combined prediction models and FS methods, as for microarrays data sets (Bolón-Canedo et al., 2014; Bommert et al., 2020), text analysis (Forman, 2003), or image interpretation (Dy et al., 2003).

In this research, several FS methods combined with linear and nonlinear learners were used to predict agronomical important traits from a high-dimensional 60K *B. napus* SNP chip data and the 15K SNP Illumina Infinium iSelect wheat genotyping platform. One major goal was to identify appropriate combinations among FS methods and linear and nonlinear learners, to decrease drastically the computing time and improve prediction accuracies for qualitative and quantitative traits. All predictions for FS-learner combinations were compared with data sets that included no SNP selection, as a benchmark. Filter methods included PCA-based data reduction and RF-based FS, while combining them with rrBLUP, LASSO, GBM, ANN, and RF learners. Similar approaches were applied in social sciences (Attewell et al., 2015), or breeding (Long et al., 2011; Montesinos-Lopez et al., 2019; Piles et al., 2021). Our results indicate that is possible to decrease the computing time up to 100 times, as for hybrid yield predictions of a *B. napus* F1 population, and also generate higher accuracies from complex traits, such as wheat grain yield under low mineral fertilizer and no fungicide treatments.

Bommert et al. (2020) investigated 16 high-dimensional data sets and compared 22 FS methods in terms of accuracy and computing time. Their findings suggested that there is no group of FS methods that outperformed all others; however, some filter methods performed much better on a specific data set. Similar results were observed also in our study. PCA data reduction combined with nonlinear learners outperformed rrBLUP and LASSO predictions. Using wheat and Jersey cows for the prediction of complex quantitative traits, Gianola et al. (2011) compared multilayer perceptron (MLP) and Bayesian ridge regression models. The authors found that the predictive Pearson's correlation in the wheat data set ranged from 0.48 with the Bayesian ridge regression, while 0.54–0.59 for MLP with one, or more, neurons. These differences were also statistically significant, improving prediction performance, between BRR and MLP, up to 18.6%. In another publication, the authors compared various types of neural networks (radial basis function neural networks and Bayesian regularized neural networks), classic linear models (Bayesian ridge regression, Bayesian LASSO, Bayes A, and Bayes B), and kernel-based models (reproducing kernel Hilbert spaces) and concluded that neural networks and kernel-based model had improved prediction accuracies in several wheat data sets (Pérez-Rodríguez et al., 2012). Similarly, Ma et al. (2018) found that neural network methods could outperform rrBLUP and GBLUP, if specific architectures, such as convolutional neural networks (CNN) or MLP, are used. The authors compared the Pearson correlation coefficient values for the prediction performed on eight important agronomic wheat traits and found that CNN had higher values than linear models, while MLP architectures underperformed. For maize, González-Camacho et al. (2012) found that kernel models [reproducing kernel Hilbert space (RKHS)] and neural networks [radial basis function neural network (RBFNN)] have similar performance in comparison with Bayesian LASSO. Also, Khaki and Wang (2019) investigated the prediction accuracies of linear methods (LASSO and regression tree) and multi-layer perception in a maize data set containing 2,267 hybrids. The results indicated that the predictions of hybrid grain yield were better with a 20 hidden layer MLP model than classical linear models.

In a recent review, the authors compared 23 independent studies in terms of linear and nonlinear prediction performance (Montesinos-López et al., 2021). The results suggested that nonlinear models outperformed linear ones in 47% of all studies (11/23), when including G × E, and in 56% (13/23), when ignoring G × E interactions. The same authors suggested that the differences could be attributed to population size, parameter tuning, or nonlinear model architecture. However, neural networks outperformed linear GS models because of their ability to better capture the structural pattern in the input data, as the CNN do. CNN are nowadays among the frequently used algorithms in plant trait analysis due to their capacity to identify complex patterns, as for root and shoot image-based feature identification (Pound et al., 2017), or to generate reliable classifications of plant stress symptoms (Ghosal et al., 2018), and wheat spikes (Hasan et al., 2018).

Machine learning methods have a high potential to become an indispensable tool for scientists and applied plant breeders. FS and modern learners can address multiple challenges as they are demonstrably powerful for integrating heterogeneous and large data sets, improve tools for higher prediction accuracies, and extracting relevant functional impacts of genotype and phenotype relationships.

Based on our approaches, the results suggested that by reducing the set of features, the computing time decreased considerably, while the accuracy of predicting was enhanced, especially for nonlinear learners. Efficient combinations among feature identification methods and modern prediction models could provide breeding programs with the necessary tools to effectively exploit cost-efficient genotyping data and improve prediction accuracies.

## Data Availability Statement

The datasets presented in this study can be found in online repositories. The names of the repositories can be found in the article/Supplementary Material. e.g. hybrid B. napus phenotype and SNP dataset in Jan et al., 2016; wheat diversity panel phenotype and SNP data in Voss-Fels et al., 2019. Supplementary Table 1 contains the parental SNP data, from Jan et al., 2016 and Supplementary Table 2 the in-silico SNP crossing for the F1 b. napus hybrids.

## Author Contributions

IG, DS, RS, and DC conceived and designed the study. IG carried out the analyses and wrote the original draft. IG and RS prepared and provided the raw data. DS, RS, and DC provided critical insights and gave methodological suggestions. All authors discussed the results, reviewed, and approved the final manuscript.

## Funding

Funding was provided by Grant No. 28DK115A20 from the German Federal Ministry of Nutrition and Agriculture (BMEL). For the Romanian authors, this work was supported by a grant of the Romanian Ministry of Education and Research, CNCS-UEFISCDI, project number PN-III-P1-1.1-PD-2019-0619, within PNCDI III and project number PN-III-P2-2.1-PED2019-0175.

## Conflict of Interest

The authors declare that the research was conducted in the absence of any commercial or financial relationships that could be construed as a potential conflict of interest.

## Publisher's Note

All claims expressed in this article are solely those of the authors and do not necessarily represent those of their affiliated organizations, or those of the publisher, the editors and the reviewers. Any product that may be evaluated in this article, or claim that may be made by its manufacturer, is not guaranteed or endorsed by the publisher.

## Supplementary Material

Supplementary Figure S Principal component analysis (PCA) of single-nucleotide polyphormism (SNP) matrix using “prcomp” in R-studio.

Supplementary Figure S2 Boxplots for the Pearson correlation, with all feature-learner methods used to select the SNPs, for seed oil content (SOC, in percentage of volume per seed dry weight) and protein content obtained in 10-outer testing sets with a ridge regression best linear unbiased prediction (rrBLUP), least absolute shrinkage and selection operator (LASSO) regression, gradient boosting machines (GBM), artificial neural networks (ANN), and random forests (RF).

Supplementary Figure S3 Boxplots for the Pearson correlation, with all feature-learner methods used to select the SNPs, for oil yield (OY, in dt/ha) and seedling emergence (SE, visual observation in a 1–9 scale) obtained in 10-outer testing sets with a rrBLUP, LASSO regression, GBM, ANN, and RF.

Supplementary Table S1 Single-nucleotide polyphormism (SNP) matrix for the parental lines, two male sterile female testers (mother lines-M) and 475 pollinators (father lines-P).

Supplementary Table S2 *In-silico* SNP matrix for the hybrid populations (F1, M × P).

Supplementary Table S3 Analysis of variance (ANOVA) and statistical tests of feature filter methods by learner combinations for hybrid seed yield (SY) and days to flower.

Supplementary Table S4 Median of the Pearson correlation over all pairs of feature scores in the 10-outer training sets obtained with different filter methods for feature selection (FS), for seed oil content (SOC, in percentage of volume per seed dry weight), oil yield (OY, in dt/ha), and seedling emergence (SE, visual observation in a 1–9 scale) and protein content.
