# Supplementary figures and images for: Machine Learning Applied to the Search for Nonlinear Features in Breeding Populations
Source: Front Artif Intell. 2022 May 20;5:876578. doi: 10.3389/frai.2022.876578 (PMC9164111; doi:10.3389/frai.2022.876578)

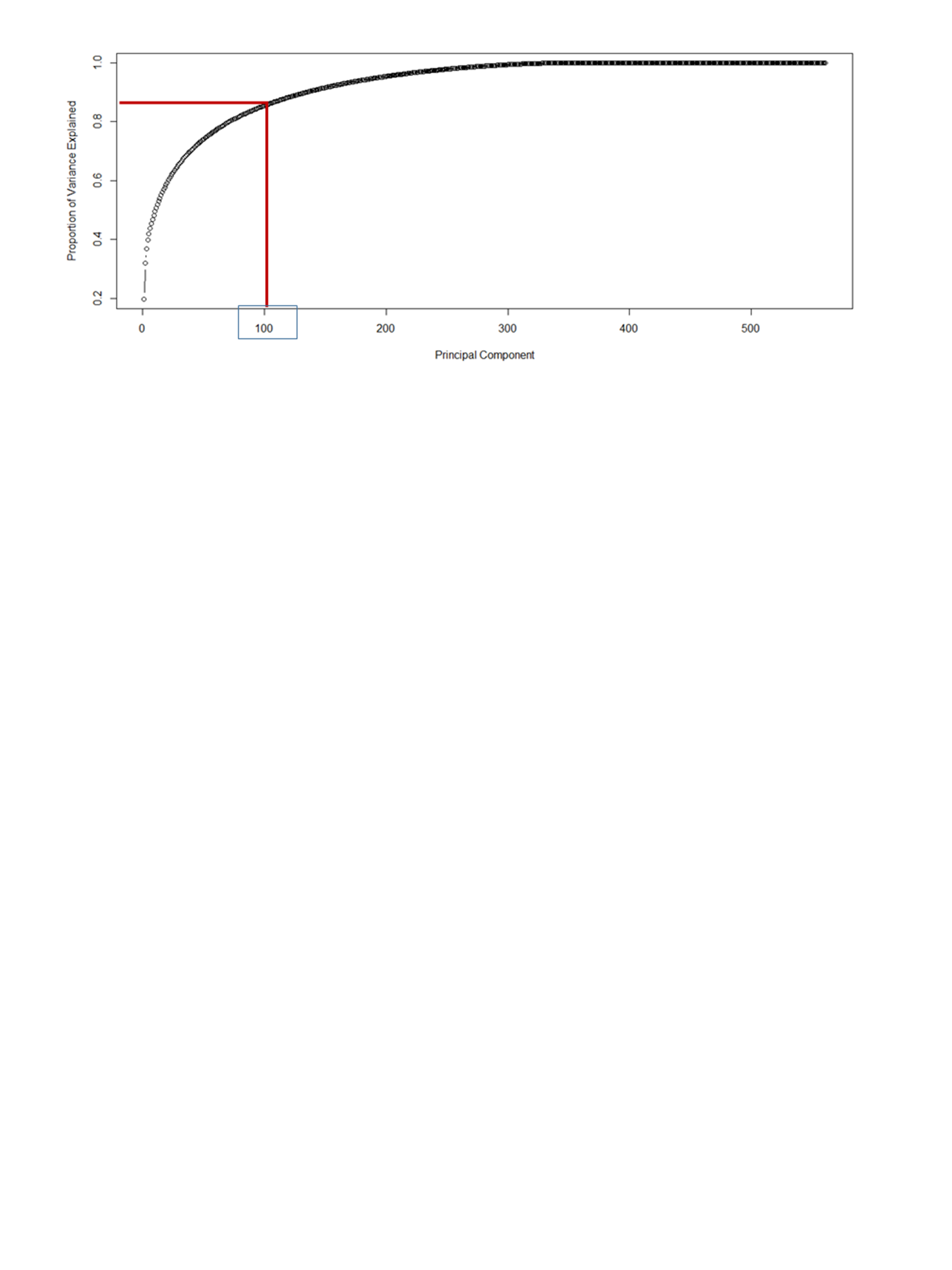

Supplement: Supplementary Figure S — Principal component analysis (PCA) of single-nucleotide polyphormism (SNP) matrix using “prcomp” in R-studio. [file Image_1.TIF]

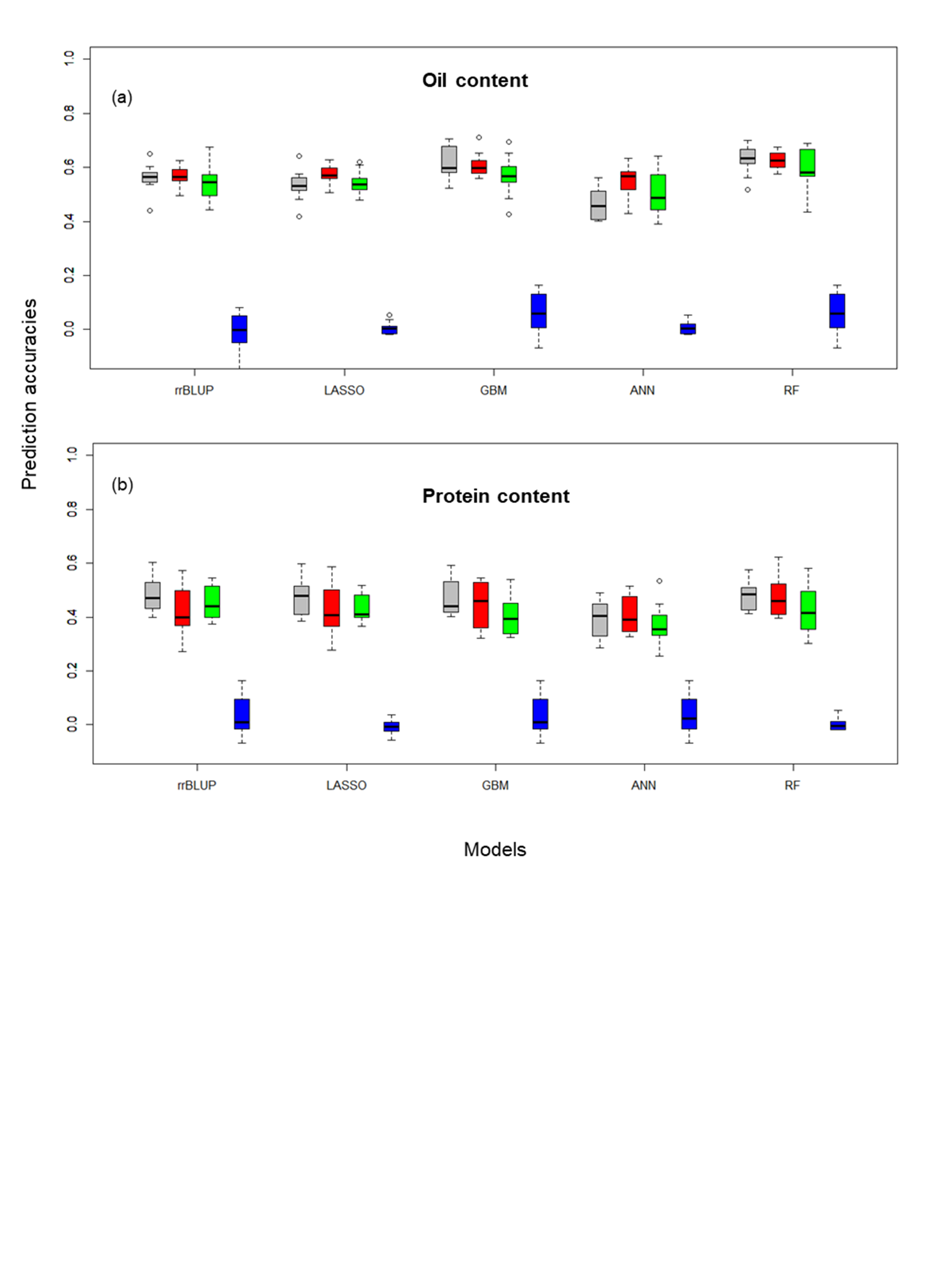

Supplement: Supplementary Figure S2 — Boxplots for the Pearson correlation, with all feature-learner methods used to select the SNPs, for seed oil content (SOC, in percentage of volume per seed dry weight) and protein content obtained in 10-outer testing sets with a ridge regression best linear unbiased prediction (rrBLUP), least absolute shrinkage and selection operator (LASSO) regression, gradient boosting machines (GBM), artificial neural networks (ANN), and random forests (RF). [file Image_2.TIF]

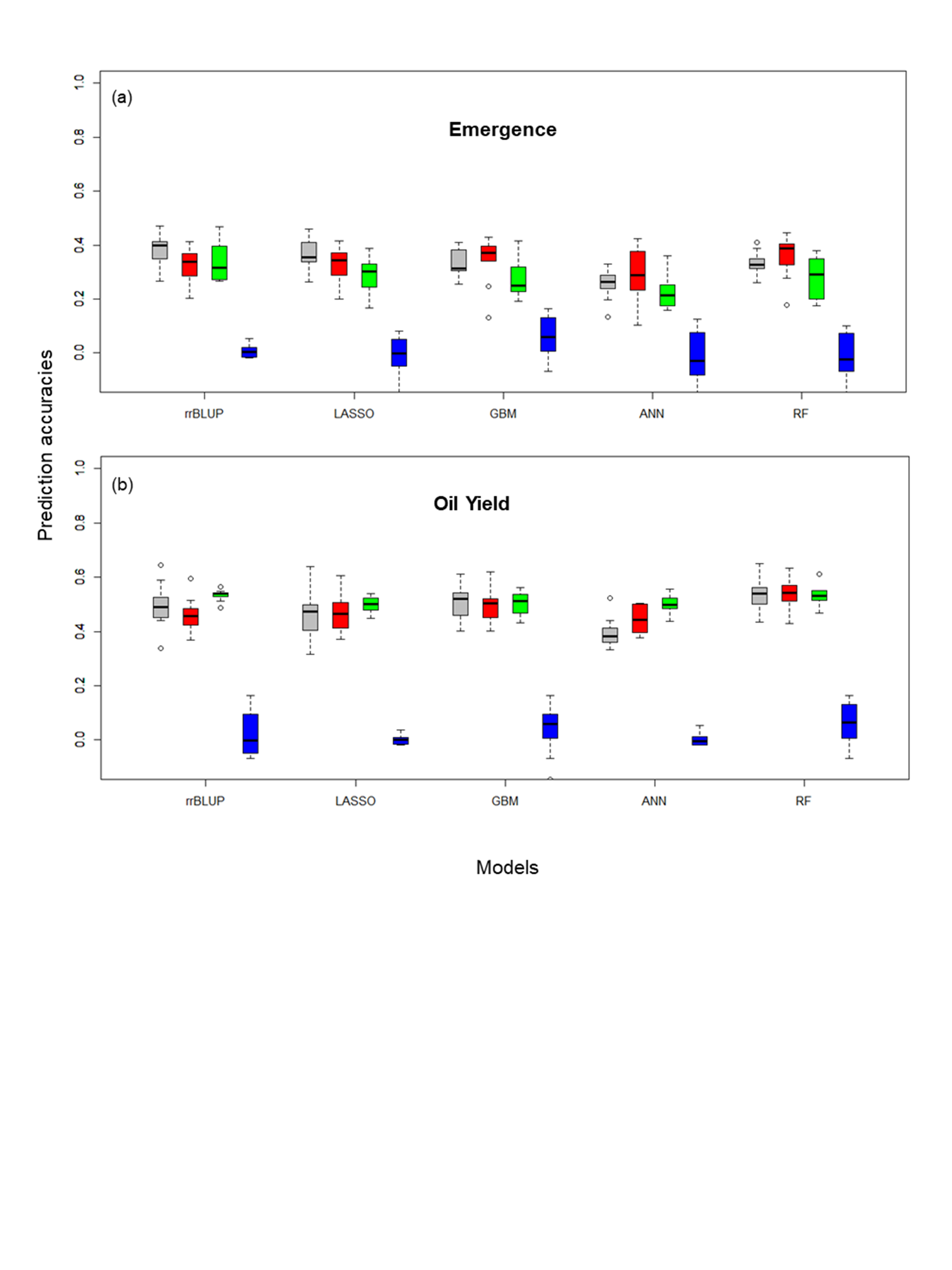

Supplement: Supplementary Figure S3 — Boxplots for the Pearson correlation, with all feature-learner methods used to select the SNPs, for oil yield (OY, in dt/ha) and seedling emergence (SE, visual observation in a 1–9 scale) obtained in 10-outer testing sets with a rrBLUP, LASSO regression, GBM, ANN, and RF. [file Image_3.TIF]
